# Characterization and genomic analysis of two *Staphylococcus aureus* bacteriophages isolated from poultry/livestock farms

**DOI:** 10.1099/vir.0.053991-0

**Published:** 2013-11

**Authors:** Hyunjin Yoon, Jiae Yun, Jeong-A Lim, Eunjung Roh, Kyu-Seok Jung, Yoonjee Chang, Sangryeol Ryu, Sunggi Heu

**Affiliations:** 1 Department of Food Technology and Services, College of Health Industry, Eulji university, Seongnam 461-713, Korea; 2 Department of Food and Animal Biotechnology, Department of Agricultural Biotechnology, Center for Food and Bioconvergence, Seoul National University, Seoul 151-921, Korea; 3 Microbial Safety Division, National Academy of Agricultural Science, Rural Development Administration, Suwon 441-707, Korea

## Abstract

*Staphylococcus aureus* is one of the most important pathogens, causing various diseases in humans and animals. As methicillin-resistant *S. aureus* (MRSA) has become increasingly prevalent, controlling this pathogen with standard antibiotic treatment has become challenging. Bacteriophages (phages) have attracted interest as alternative antibacterial agents to control MRSA. In this study, we isolated six *S. aureus* phages from soils of poultry/livestock farms. Based on the results of host range determination with 150 *S. aureus* strains and restriction enzyme treatment of phage DNA, two phages, designated SP5 and SP6, were selected for further characterization and genome sequencing. Both SP5 and SP6 were classified as members of the family *Siphoviridae*. The genome of SP5 comprises 43 305 bp and contains 63 ORFs, while the SP6 genome comprises 42 902 bp and contains 61 ORFs. Although they have different host spectra, the phage genomes exhibit high nucleotide similarity to each other. Adsorption assay results suggested that the host range determinants of the two phages are involved in both adsorption and infection. Comparative genomic analyses of the two phages provided evidence that the lysogenic/lytic control module and tail proteins may be important for host specificity.

## Introduction

*Staphylococcus aureus* is usually commensal on human skin, but it is one of the most important pathogens, causing illnesses ranging from minor skin infections to life-threatening diseases such as pneumonia and bacteraemia (Mann, 2008). As well as humans, livestock animals are also infected with *S. aureus*, resulting in bovine mastitis and chicken arthritis, which threaten food safety and cause food poisoning (Vanderhaeghen *et al.*, 2010). Notably, the increasing incidence of antibiotic-resistant *S. aureus* strains, such as methicillin-resistant *S. aureus* (MRSA) and vancomycin-resistant *S. aureus* (VRSA), has become a major concern, because *S. aureus* is one of the most common causes of nosocomial infections (DeLeo & Chambers, 2009; Dulon *et al.*, 2011).

*S. aureus* bacteriophages have been investigated extensively. All sequenced *S. aureus* bacteriophages are grouped into three classes based on genome size: class I (<20 kb), class II (~40 kb) and class III (>125 kb) (Kwan *et al.*, 2005). Bacteriophages belonging to class III have been characterized as members of the family *Myoviridae* and are obligate lytic phages with relatively wide host ranges (Kwan *et al.*, 2005; Mann, 2008). Obligate lytic phages have received renewed interest as potential therapeutic agents to replace or supplement antibiotics in the treatment of MRSA or other antibiotic-resistant *S. aureus* strains. Clinical trials performed in Eastern Europe showed that bacteriophage therapy was effective in the treatment of many *S. aureus*-related diseases, including lung infection, sepsis and leg ulcers (Kutateladze & Adamia, 2010; Rhoads *et al.*, 2009). Temperate phages, on the other hand, have been regarded to be pathogenically important because they often carry virulence genes in *S. aureus*. For example, phage 80α can mobilize a variety of superantigen-encoding pathogenicity islands (SaPIs) (Tallent *et al.*, 2007), and another major group of virulence factors, the Panton–Valentine leukocidin genes, are encoded by prophages such as φPVL and φSLT (Narita *et al.*, 2001; Zou *et al.*, 2000).

In this paper, we report isolation of *S. aureus* bacteriophages from soil samples from a poultry farm and a cattle shed, and present the results of microbiological and molecular characterization of two selected phages that can be utilized in developing biocontrol agents against staphylococcal contamination.

## Results and Discussion

### Isolation of bacteriophages infecting *S. aureus*

Bacteriophages infecting *S. aureus* are potential biological control agents that can prevent staphylococcal food poisoning. In order to apply isolated bacteriophages to control *S. aureus* infection by contaminated foods in human and livestock, we first isolated and characterized phages with strong lytic activity against *S. aureus* strains. Six *S. aureus* bacteriophages were isolated from six soil samples collected from poultry and livestock farms, and designated SP1–SP6. Based on host specificities and restriction enzyme digestion profiles, these six phages were categorized into two groups. SP5 and SP6 were selected as representatives of these two groups and characterized in depth.

### Host specificity of six phages

The host specificities of the six isolated phages were determined using 10 *S. aureus* reference strains and 140 isolates originated from humans, livestock, vegetables and foods (Table 1). SP1, SP2, SP4 and SP5 (SP5-like phages) showed identical host specificities, lysing 29 isolates (20.7 %); SP3 and SP6 (SP6-like phages) shared different host specificities, lysing 33 of 140 isolates (23.6 %). Only 18 isolates were sensitive to both phages. None of the six phages could lyse other bacterial species, including *Staphylococcus epidermidis* [ATCC 35983, Culture Collection of Antimicrobial Resistant Microbes (CCARM) 3787 and 3789], *Listeria monocytogenes* (ATCC 19115), *Bacillus subtilis* (ATCC 23857), *Bacillus cereus* (ATCC 14579), *Pseudomonas aeruginosa* (ATCC 27853) and *Escherichia coli* (MG1655, ATCC 35150) (data not shown).

**Table 1.** Lytic activity of isolated phages towards *S. aureus* strains

Strains tested	SP1, SP2, SP4 and SP5	SP3 and SP6
ATCC 29213, CCARM 3089 and 18 isolates	**+**	**+**
CCARM 3090 and 11 isolates	**+**	**–**
15 isolates	**–**	**+**
ATCC 25904 (Newman), ATCC 12600, ATCC 25923, ATCC 6538, ATCC 23235, ATCC 27664, CCARM 3793 and 96 isolates	–	–

### Restriction enzyme digestion profiles

**Fig. 1.**
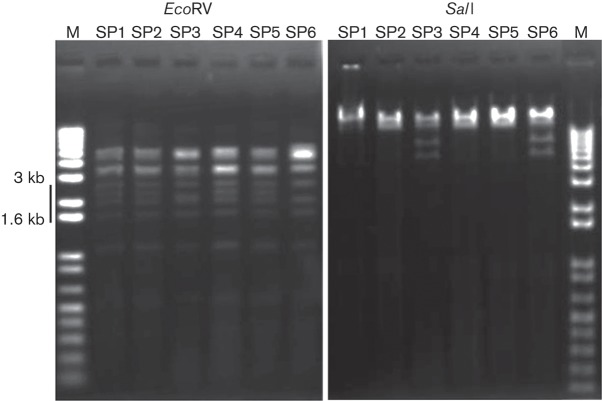
Restriction enzyme digestion of six phage DNA samples. The patterns on 1 % agarose gels following *Eco*RV digestion (1.6–3 kb) and *Sal*I digestion differed between SP5-type phages (SP1, SP2, SP4 and SP5) and SP6-type phages (SP3 and SP6). M, Molecular marker.

For genotyping analysis between the isolated phages, genomic DNA was extracted from the six phages and treated with various restriction enzymes. Comparison of the restriction fragments by agarose gel electrophoresis showed that SP1, SP2, SP4 and SP5 shared the same profile, while SP3 and SP6 had an alternative profile (Fig. 1).

### Phage morphologies

**Fig. 2.**
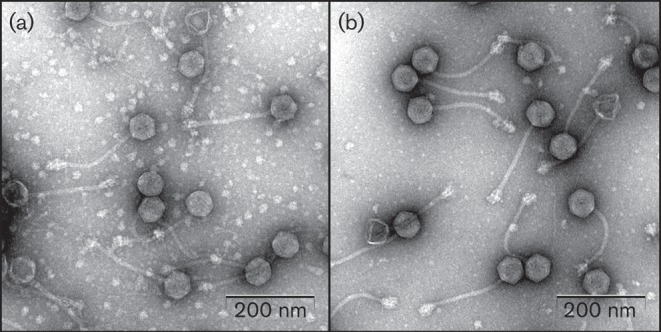
Electron micrographs of phages SP5 (a) and SP6 (b).

All the isolated phages were visualized by transmission electron microscopy (TEM) (Fig. 2). All phages exhibited hexagonal heads (diameter ~60 nm) and non-contractile, flexible tails (length ~200 nm), which are representative features of bacteriophages belonging to the family *Siphoviridae* (order *Caudovirales*). The tail of each phage contained a baseplate with spikes at the end.

### Differences between SP5 and SP6 in adsorption to their hosts

Based on the results of host range determination and genotyping analyses, SP5 and SP6 were chosen for further studies below. The difference in host specificity between SP5 and SP6 may be attributable to bacterial host determinants such as receptors as well as phage features. Interestingly, the 11 bacterial isolates sensitive only to SP5-like phages were human- or livestock-derived, suggesting the possibility of the presence of common genetic features among these 11 isolates. To gain insight into the host range determination, multiple analytical tools including diagnostic PCR and pulsed-field gel electrophoresis (PFGE) were applied to *S. aureus* isolates to examine their genetic relationships and virulence factors. Strong genetic diversity was high among bacteria isolates; there was little correlation between host genetic diversity and phage host range determination (data not shown).

**Fig. 3.**
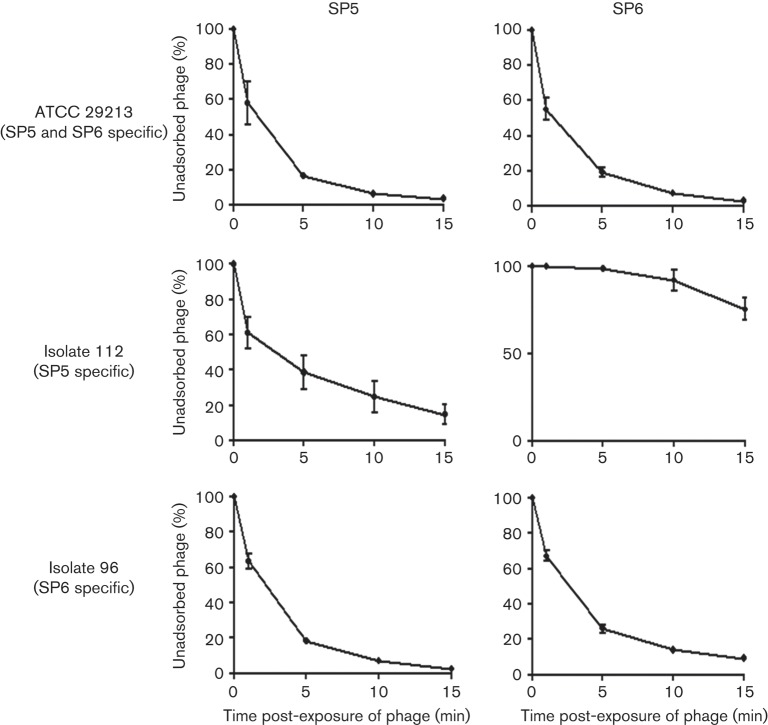
Adsorption of SP5 and SP6 to their host strains. *S. aureus* ATCC 29213 was sensitive to both phages, while human isolates 112 and 96 were only susceptible to SP5 and SP6, respectively. Data are presented as the means±sd of triplicate assays.

In order to find evidence to explain the difference in host specificity between SP5 and SP6, an adsorption assay was performed (Fig. 3). Both phages adsorbed efficiently to their common host, *S. aureus* ATCC 29213, as expected. For *S. aureus* isolate 112, which could be infected by SP5 only, the adsorption affinity of SP5 was higher than that of SP6, suggesting that SP6 phage might be lacking the ability to attach to the host surface. However, for isolate 96, which is sensitive to SP6 only, the adsorption affinity of SP5 was comparable to that of SP6, suggesting that SP5 might fail in intracellular replication or escaping the host cell after invasion. These adsorption assay results indicate that host range determinants are associated not only with the adsorption stage (isolate 112) but also with the stage after adsorption (isolate 96) (Fig. 3).

### Genome overviews of SP5 and SP6 phages

For a better understanding of the different host specificity among phages, genomic comparison between SP5 and SP6 was carried out. The genome of SP5 comprised 43 305 bp and contained 63 putative ORFs (Table S1, available in JGV Online); the genome of SP6 comprised 42 902 bp and contained 61 putative ORFs (Table S2). The G+C contents of SP5 and SP6 were 34.4 and 34.5 mol%, respectively, slightly higher than that of the sequenced *S. aureus* strains (32.8 %). Neither phage seemed to contain tRNA-coding regions, according to tRNAscan-SE v.1.21 analysis. Neither known virulence factors, including staphylokinase, exfoliative toxin A, enterotoxin A, Panton–Valentine leukocidin, nor the innate immune modulators CHIPS (chemotaxis inhibitory protein of *S. aureus*) and SCIN (staphylococcal complement inhibitor), were found in either SP5 or SP6 (Betley & Mekalanos, 1985; Narita *et al.*, 2001; van Wamel *et al.*, 2006; Winkler *et al.*, 1965; Yamaguchi *et al.*, 2000).

**Fig. 4.**
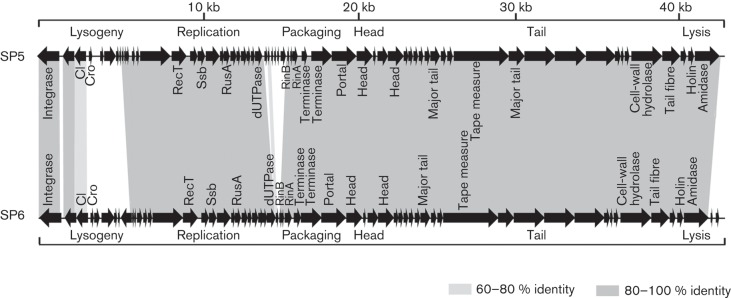
Genomic maps of phages SP5 and SP6. Predicted ORFs are denoted by arrows, and genes encoding proteins with at least 60 % amino acid identity between the two genomes are indicated by shaded regions.

The overall genome organizations of SP5 and SP6 are compared in Fig. 4. All of the ORFs, except for the first three, are transcribed rightward in both genomes. Both genomes are organized into three major gene clusters: (i) the ‘lysogeny’ control region; (ii) the ‘early’ region, encoding products involved in replication, recombination and modification of the phage DNA; and (iii) the ‘late’ region, encoding structural and assembly proteins and lysis proteins.

blastn analyses revealed that SP5 and SP6 had high nucleotide similarity with other known *S. aureus* phages including 80α (GenBank accession no. DQ517338), φ11 (GenBank accession no. AF424781), 53 (GenBank accession no. AY954952) and 69 (GenBank accession no. AY954951) phages. The query coverage was from 65 to 73 % and homologous sequences were conserved at the latter parts of SP5 (>12 kb) and SP6 (>12.5 kb) genomes. Interestingly, both phages also exhibited high sequence similarity with the φ37-like prophage region of *S. aureus* 11819-97 (GenBank accession no. CP003194) with 89–91 % query coverage (Stegger *et al.*, 2012) (Table S3).

### Comparative genomics of SP5 and SP6

SP5 and SP6 bacteriophages appeared to be temperate, rather than virulent, phages belonging to class II *S. aureus* phages (Kwan *et al.*, 2005) based on genome analyses but also had strong lytic activities showing ~10^6^-fold reduction in bacterial number for 3 h infection (data not shown). Genome sequencing of SP5 and SP6 revealed that these two phages shared the highest sequence similarity to each other (query coverage >90 %), albeit there were also other phages or prophages with high nucleotide similarity. Among the 63 ORFs of SP5 and 61 ORFs of SP6, 53 ORFs were highly conserved in both phages: 32 ORFs had 100 % identities; 19 ORFs had >90 % identities; and two ORFs had >70 % amino acid identities (Fig. 4, Table S4). Comparative genomic analysis between the two phage genomes provided deeper insights into their characteristics including the different host spectra.

### The ‘lysogeny’ control region

Comparing the phage genomes of SP5 and SP6, the most divergent part lay in the ‘lysogenic’ control region. Although the first three ORFs (integrase to CI protein) showed high identities (76–100 %) between the two genomes, the subsequent ORFs (Cro-like protein to orf8 in SP5/orf9 in SP6) showed no such similarities to each other (Tables S3 and S4). The functions of many ORFs in this divergent region are unknown, but this region appears to be important for regulation of phage lysis/lysogeny because it includes proteins such as Cro-like repressor, phage antirepressor (orf6 in SP5 and SP6), and a helix–turn–helix domain-containing protein (orf5 in SP5). While CI is the regulatory protein that stabilizes the lysogenic state, Cro makes lytic growth and the lytic pathway possible by repressing CI (Little, 2007). An antirepressor protein (orf6) of SP5 and SP6 may be responsible for inactivation/bypass of the CI transcription repressor (García *et al.*, 2009). In addition, the nucleotide sequences of the intergenic regions between *cI* and *cro*, which contain promoter regions for *cI* and *cro*, are totally different. This indicates that the mechanisms regulating the switch between the lysogenic and lytic states may differ between the two phages, and may be affected by different signals or regulator proteins.

Computer-aided promoter prediction (http://linux1.softberry.com/berry.phtml) of the intergenic space between the *cI* and *cro* genes suggested differential transcriptional regulation by a variety of regulators in the two phages (Fig. S1). Features included consensus RpoD-binding sites upstream of *cI* and *cro* in SP6 and *cI* in SP5, but not in the upstream region of *cro* gene in SP5, and an Lrp-binding site upstream of *cro* in SP5. This computational inference suggests the possibility that the transcription of *cro* in SP5 by σ70 may be repressed by Lrp, and that alternative regulatory factors may be required to induce the lytic pathway in the host. Lrp, a global regulator, interferes with open complex formation in σ70- or σS-containing RNA polymerase holoenzymes at promoters and inhibits the initiation of transcription (Colland *et al.*, 2000). Thus, this difference in the lysogenic/lytic regulation module may be the determinant of the different host specificities between SP5 and SP6, and may explain why phage SP5 could not lyse *S. aureus* isolate 96, despite equivalent adsorption affinities for isolate 96 between the two phages, although this possibility needs to be tested (Fig. 3).

### The ‘early’ region

The regions from orf9 to orf32 in SP5 and from orf10 to orf28 in SP6 appeared to be included in the ‘early’ region. This region seemed to contain genes related to DNA replication and metabolism. Included in this region are the DNA recombination protein RecT (orf15 in SP5 and orf16 in SP6), the ssDNA-binding protein Ssb (orf17 in SP5 and orf18 in SP6), the crossover junction endoDNase RusA (orf20 in SP5 and orf21 in SP6) and dUTP diphosphatase (orf26 in SP5 and orf27 in SP6), all of which appeared to function during DNA recombination. All the ORFs belonging to this region were conserved in the genomes of SP5 and SP6, with extremely high amino acid identities. However, five ORFs (orf27 and orf29–orf32) were found only in the genome of SP5. These ORFs have high similarity to proteins belonging to *S. aureus* subsp. *aureus* Mu50. However, the functions of these five ORFs remain unknown.

### The ‘late’ region

This region contains the structural and lysis modules. The structural module can be divided into packaging, head, head–tail connection and tail submodules. This region starts from *rinB* (orf33 in SP5 and orf29 in SP6) and ends at the termini of the genomes. As shown in Fig. 4, all ORFs in the ‘late’ region were conserved in both genomes, with high amino acid sequence identity (>96 %), except the rightward accessory region (orf60 and orf61 in SP6), which was unique to the genome of SP6.

This region starts with the *rinAB* regulator genes. RinA is reported to be the major regulator of phage-mediated packaging, capsid assembly and cell lysis in phage 11, whereas RinB does not appear to control the expression of ‘late’ genes (Ferrer *et al.*, 2011). Among ORFs belonging to the structural module, orf59 of SP5 and orf55 of SP6 were annotated as tail-associated cell-wall hydrolases. Tail-associated cell-wall hydrolase is localized at the tail tip and allows contact with the cell during adsorption/DNA injection in phage infection (Rashel *et al.*, 2008). However, the tail-associated cell-wall hydrolases from SP5 and SP6 had 100 % identity to each other, ruling out the possibility of tail tip hydrolase being responsible for host specificity.

To date, no report on determinants of host specificity of *S. aureus* phages has been published. However, with respect to other phages, the best-known determinant is the tail tip of phages, by which bacteriophages recognize receptors and inject DNA (Letellier *et al.*, 2007; Tétart *et al.*, 1996; Werts *et al.*, 1994). Since the adsorption affinities of two phages were clearly distinguished by an SP5-specific host (isolate 112) (Fig. 3), we considered the possibility that tail fibre proteins may control host spectra. When the amino acid sequences of tail fibre proteins were compared among phages SP5 and SP6, and another 14 phages with tail fibre proteins of similar sizes (~390 aa) from *Staphylococcus* prophages and staphylococcal phages in the family *Siphoviridae*, moderate sequence diversities at four loci in the C-terminal region were found (each locus ~20 aa), whereas N-terminal portions (the N-terminal 240 aa) were highly conserved among 16 tail fibre proteins (Fig. S2). Host specificity among *Staphylococcus* phages may be attributable to the sequence diversity in the C-terminal portion of tail fibre proteins, which is implicated in binding to host receptors. Enterobacteria phage λ utilizes tail fibre protein J in binding to the LamB receptor on the outer membrane of *E. coli* and the C terminus of tail fibre protein J (20 % distal C-terminal portion) was found to be important for the interaction of the phage with the host receptor protein *in vitro* and *in vivo* (Hofnung *et al.*, 1976; Werts *et al.*, 1994).

The lysis modules of both phages comprise the canonical holin–endolysin cassette. The amino acid sequences of the holin and endolysin proteins of SP5 (orf62 and orf63) and SP6 (orf58 and orf59) exhibited 96 % identities. These endolysin proteins harbour a CHAP (cysteine, histidine-dependent amidohydrolases/peptidases) (PF05257, cysteine peptidase) domain and an amidase (PF01510) domain as catalytic domains at the N terminus, and an SH3_5 (PF08460) domain for substrate-binding at the C terminus.

### Conclusion

In conclusion, our data demonstrated that two *S. aureus* siphoviruses isolated from a poultry farm and a cattle shed have high similarity in their genomic sequences but different host specificities. Comparative genomic analyses of these phages suggested the possibility that the difference in host specificities might be caused by different lysogenic/lytic control mechanisms and different tail fibre proteins between the two phages. Although further studies to support this conclusion are necessary, the results described here improve our understanding of the genomes of *S. aureus* phages.

## Methods

### 

#### Bacterial strains, growth conditions and bacteriophage methods.

The bacterial strains were acquired from the American Type Culture Collection (ATCC) and CCARM (Culture Collection of Antimicrobial Resistant Microbes, Seoul, Republic of Korea). *S. aureus* ATCC 29213 was routinely used to propagate isolated bacteriophages after purification and to determine the titres of phage stocks. Other strains used for host range determination are shown in Table 1 or are described in Results and Discussion. One hundred and forty *S. aureus* isolates were obtained from the collection of the Rural Development Administration (Suwon, Republic of Korea). These isolates originated from humans (37 isolates), livestock and their products (40 isolates), vegetables (54 isolates) and gimbap (Korean rice rolled in laver paper) (nine isolates).

All bacterial strains were routinely cultivated at 37 °C using tryptic soy broth (TSB) or tryptic soy agar (TSA) (Difco). CaCl_2_ (10 mM) and MgCl_2_ (10 mM) were added when bacteriophages were propagated. Spotting and overlay assays were routinely performed to form plaques and determine phage titres using TSA plates (0.5 % agar for the top, 1.5 % agar for the bottom) containing CaCl_2_ (10 mM) and MgCl_2_ (10 mM) according to methods described previously (Kronpinski *et al.*, 2009; Mazzocco *et al.*, 2009). SM buffer [50 mM Tris/HCl (pH 7.5), 100 mM NaCl, 10 mM MgSO_4_] was used to dilute phage stocks. Plaque formation was verified after incubation at 37 °C for 16 h.

#### Isolation of bacteriophages.

Six soil samples were collected from a poultry farm and a cattle shed. Bacteriophages were isolated as described previously (Kim & Ryu, 2011). Briefly, samples (25 g) were homogenized in 25 ml homogenization buffer (0.25 M KH_2_PO_4_ adjusted to pH 7.2 with NaOH) for 90 s in a BagMixer 400 blender (Interscience Laboratory). Initial propagation was performed with the filtrate of each homogenized sample and a mixture of overnight cultures of 140 *S. aureus* isolates. Bacteriophages were isolated and purified by overlay assays (repeated three times) by picking plaques and elution in SM buffer.

#### Propagation and purification of bacteriophages.

*S. aureus* ATCC 29213 was employed to propagate bacteriophages routinely. Bacteriophage particles were precipitated with polyethylene glycol 6000 (10 %) and NaCl (1 M) at 4 °C overnight. After centrifugation (10 000 ***g***, 20 min, 4 °C), precipitated bacteriophages were resuspended in SM buffer, and purified by CsCl density gradient ultracentrifugation (78 500 ***g***, 2 h, 4 °C). Separated bacteriophages were dialysed against 1000 vols TM buffer (50 mM Tris/HCl (pH 7.5), 20 mM MgSO_4_) for 2 h with one buffer change. The titre of each purified bacteriophage was determined by overlay assay (Kronpinski *et al.*, 2009). Phage stocks were stored in glass tubes at 4 °C.

#### Host range determination.

The host range of each phage was determined by spot test. Lysis zone or plaque formation was monitored upon application of 10 µl of each phage lysate, adjusted to contain 1×10^7^ p.f.u. ml^−1^, by spotting assay.

#### Bacteriophage DNA extraction and restriction.

Phage DNA was extracted by an alkaline lysis method (Wilcox *et al.*, 1996). Phage DNA (1 µg) was digested with 20 U restriction enzymes (Takara Biomedical) for 16 h according to the manufacturer’s instructions, and was resolved in a 1 % agarose gel.

#### TEM.

Purified bacteriophages were visualized by TEM. TEM was conducted as described previously (Kim & Ryu, 2011). Based on their morphology, phages were identified and classified according to the guidelines of the International Committee on Taxonomy of Viruses (Fauquet *et al.*, 2005).

#### Genome sequencing and *in silico* analysis.

Genomic DNA was isolated from phages SP5 and SP6 by the alkaline lysis method (Wilcox *et al.*, 1996). A pyrosequencing approach, using a Genome Sequencer FLX System (Titanium series; Macrogen) was applied, and the high-quality filtered reads were assembled into a complete genome sequence using GS *De novo* Assembler (v.2.6). ORFs were predicted with Glimmer 3 (Delcher *et al.*, 2007), GeneMarkS (Besemer *et al.*, 2001) and FgenesB (http://linux1.softberry.com/berry.phtml). RBS finder (Suzek *et al.*, 2001), which facilitates determination of the start codon of each ORF, was used to predict ribosome-binding sites. Annotation of predicted ORFs was performed by analysing the results of a similar protein search using blastp (Altschul *et al.*, 1990) and a motif search using InterProScan (Quevillon *et al.*, 2005). A search for tRNA-coding regions was performed using tRNAscan-SE v.1.21 (Schattner *et al.*, 2005).

#### Adsorption assay.

Adsorption of the phages was assayed as follows. Overnight cultures of the bacterial strains were diluted 1 : 100 in tryptic soy broth (TSB). When the OD of the reference strains at 600 nm reached 2.0, 1 ml of each culture was diluted 10-fold in fresh TSB. SP5 and SP6 phages were added at an m.o.i. of 0.01 to each diluted culture, which contained 10 mM CaCl_2_ and 10 mM MgCl_2_, mixed gently, and incubated at 37 °C for 15 min. An aliquot (100 µl) was removed immediately for determination of the initial phage titre. Incubation was continued for 15 min, and samples (100 µl) were collected after 1, 5, 10 and 15 min and diluted immediately in 900 µl cooled SM buffer. The diluted samples were centrifuged at 14 000 ***g*** for 1 min at 4 °C and filtered using 0.22 µm filters. Finally, the titres of unabsorbed phages in the supernatant were determined after serial dilution.
